# Improving hospital food and meal provision: a qualitative exploration of nutrition leaders’ experiences in implementing change

**DOI:** 10.1186/s12913-025-12499-x

**Published:** 2025-03-19

**Authors:** Emma Wilandh, Malin Skinnars Josefsson, Christine Persson Osowski, Ylva Mattsson Sydner

**Affiliations:** 1 Department of Food Studies, Nutrition and Dietetics, Uppsala University, Box 560, Uppsala, 751 22 Sweden; 2 Division of Public Health Sciences, School of Health, Care and Social Welfare, Mälardalen University, Box 883, Västerås, 721 23 Sweden

**Keywords:** Hospital, Foodservice, Meals, Patient, Implementation, Improvement, Qualitative interviews, Thematic analysis, Facilitation, Leadership

## Abstract

**Background:**

Recently, numerous initiatives have been taken to improve food and meals for hospital inpatients. Research providing in-depth knowledge on leading such improvement initiatives and implementing changes, specifically through facilitation within this multilevel context, is essential. This study aims to explore nutrition leaders’ experiences in implementing changes to improve food and meal provision for hospital inpatients, focusing on facilitation activities.

**Method:**

This is a qualitative interview study within the social constructivist paradigm. Participants were recruited through professional networks, advertisements, and snowballing. Eighteen semi-structured interviews were conducted individually with participants in leadership roles of food and meal improvement initiatives at Swedish hospitals. The interviews were transcribed verbatim and analysed thematically through an i-PARIHS lens.

**Results:**

Three themes of facilitation activities were identified: ‘Building Relationships’, ‘Placing Food and Meals on the Agenda’, and ‘Cultivating Skills’. Building relationships involved establishing connections between the service and clinical divisions. Creating common structures and multidisciplinary teamwork enabled collaboration across organisational boundaries. Placing food and meals on the agenda involved both initial and ongoing communication activities, as food and meal tasks were often considered low priority. Cultivating skills encompassed creating learning opportunities for implementing lasting changes, tailored to specific contexts and adopted within everyday practices.

**Conclusions:**

Collaboration between foodservice and clinical professionals, along with the dissemination of knowledge, appears to be important for implementing changes. Active leadership supports successful implementations by providing structured approaches, including feedback systems, and by contributing to the recognition of improvement initiatives, according to experiences shared during interviews.

## Background

Appropriate hospital food and meal provision is fundamental to clinical nutritional care [1] and crucial for positive patient outcomes [2, 3]. Nevertheless, the standard of food and meals for inpatients has long been criticised for various reasons targeting multiple different aspects. Foremost critics are in relation to undernutrition. Poor food and meal intake is reported by hospitals worldwide [4], contributing to worsened patient outcomes, increasing the incidence of undernutrition, and prolonging hospital stays [5, 6]. Hence, recently, numerous improvement initiatives have been taken to enhance food and meal standards, thereby improving patients’ intakes [7, 8]. For example, efforts have focused on optimising menu offerings and diet quality [9, 10], as well as the flexibility [11, 12] and frequency of meals [13, 14]. However, shortcomings in both organisation and leadership frequently hamper improvements [15, 16], as pointed out decades ago in a European resolution [17]. In this resolution on food and nutritional care in hospitals, the identified shortcomings include unclear responsibilities and a lack of interaction between the numerous actors involved, making it difficult to coordinate decisions. These actors represent healthcare and service staff and professionals, politicians, and patients. Furthermore, competence in relation to the food served and the hospital meal situation has been identified as problematic [18, 19].

Hospitals are highly multidisciplinary and hierarchical organisations, considered complex multilevel contexts that pose challenges regarding the implementation of changes for improvements [20–22]. This complexity arises from the presence of varying perspectives, both between different professional groups and among various departments, divisions, and organisational levels within the hospitals, as these represent different contexts with various resources and priorities. Implementing changes to improve the provision of food and meals for hospital inpatients appears particularly challenging. This is due to the several interconnected and multifaceted tasks that require broad engagement and collaboration among various professionals and staff groups. These include those in clinical care, such as assistant nurses and nurses, as well as foodservice and allied health professionals, who are involved in nutritional care, meal ordering and service. These professionals and staff have different types of experience, educational backgrounds, and diverse competencies within their individual disciplines. Establishing collaboration among different professionals, who approach food and meals from various perspectives, and getting them to work jointly towards a shared goal of implementing changes seems challenging. However, multidisciplinary collaboration, including patient involvement, is required for food and meals to be planned, produced, ordered, delivered, served, and eaten by the patient [23–26]. Moreover, in improvement initiatives within the provision of food and meals for hospital inpatients, effective implementation requires the involvement of professionals with relevant skills [27–29], a supportive structure with clear roles and context-specific activities [30], and a demonstration of active leadership [31–33]. Adding to the complexity, implementing changes regarding food and meal tasks requires the involvement of professionals from different disciplines and hierarchical levels within the hospital [34–36].

Globally, research exploring how to implement change within this field is limited [37, 38], although several national and expert guidelines exist to support improvements in the provision of food and meals for inpatients [39–42]. In 2020, the Swedish Food Agency published national guidelines for hospital meals, followed in 2022 by a broad mapping of the different kinds of improvements implemented in hospitals across the country [43]. However, there is a research gap concerning how improvement initiatives are carried out, particularly regarding the facilitation of implementing changes. There is also a lack of qualitative investigations that provide a deeper understanding of this. Additionally, improvement initiatives within the food and meal provision for hospital inpatients have primarily focused on systemic changes, while professional relationships and the leadership role in this context have received limited attention [44]. Greater in-depth knowledge of the multilevel complexity and associated challenges can provide valuable insights and examples of how to implement changes for improvement and the facilitation of these initiatives. Facilitation activities, ranging from specific tasks such as providing instructions to overarching supportive encouragement, are defined according to a description from Harvey et al. (p. 580) on facilitation as ‘the process of enabling (making easier) the implementation of evidence into practice’ [45]. As such, ‘facilitation activities’ are thought to support change and implementation success.

Therefore, more qualitative research involving individuals in leadership roles in implementing changes and related facilitation activities within improvement initiatives is needed. Evidence-based knowledge is essential for healthcare providers in guiding policy formulation and shaping future practices. Consequently, this study aims to explore nutrition leaders’ experiences in implementing changes to improve food and meal provision for hospital inpatients, focusing on facilitation activities.

## Methods

This is a qualitative interview study within the social constructivist paradigm [46]. A qualitative approach is particularly suitable for capturing detailed and comprehensive experiences [47]. A social constructivist paradigm entails an ontological stance of multiple realities being constructed through experiences and interactions with others, as well as an epistemological view that knowledge is influenced by individuals’ experiences in a specific social and historical context [46]. The focus of the study was to explore participants’ experiences as nutritional leaders in hospital improvement work in Sweden. The study design was further guided by the integrated Promoting Action on Research Implementation in Health Services (i-PARIHS) framework [48] to ensure that all aspects of implementing changes were included. Facilitation is central to both this framework and this study, as key dimensions in the i-PARIHS framework include the facilitator role, assumed either by an individual or a group, and facilitation entailing several activities. Additionally, contextual support at multiple levels is central to i-PARIHS, ranging from the inner context, including recipients (those most affected by the changes) and the innovation itself (the novelty to be implemented), to the outer context comprising decision-makers at all levels, including politicians. The entire context needs to be considered for achieving lasting changes [49]. The framework has been used in the context of food and meal improvement initiatives within healthcare [50, 51], making it suitable for this study. It served as a lens for both constructing the interview guide and analysing the interviews.

### The research setting and context

To provide context for international readers, it’s important to understand the structure of the Swedish healthcare system. The system comprises twenty-one self-governed Regions, each including one or multiple hospitals. The provision of food and meals for inpatients within these hospitals is ultimately governed by regional-level politicians and is a tax-funded operation. The hospital’s service division is responsible for the foodservice department, regardless of whether the food and meals are publicly or privately procured. A central kitchen may exist within the hospital or at a remote location serving multiple hospitals. The service division may have employees stationed in the ward kitchen performing tasks such as plating, organising trays, and reheating food and meals; if not, these tasks fall to the clinical division. Collecting food orders, serving meals, and attending to patients during mealtimes are tasks that typically fall under the remit of the clinical division and thus their employees’ responsibility.

### The recruitment

The recruitment process was initiated by contacting a purposive sample of individuals leading improvement initiatives within the provision of food and meals for hospital inpatients and, consequently, engaged in implementing associated changes. To capture valuable experiences, the nutrition leaders needed to be individuals who actively manage and are highly committed to this work in Swedish hospitals. This was achieved through the professional networks of the research team members. The process continued by advertising for additional nutrition leaders, i.e., individuals in management positions or other leadership roles involved in relevant improvement initiatives. These advertisements were placed in food and nutrition profession-specific social media groups, targeting professionals within our scope of research. Once the first set of participants had been interviewed, the recruitment method shifted from purposive to snowball sampling; participants were encouraged to recommend other suitable interview candidates. Recruitment, concurrent with data collection and analysis, continued until data saturation was reached, indicating that no new substantial information was being uncovered during the interviews [52]. Although data saturation is discussed as a problematic concept and difficult to establish in qualitative research [53], a considerable amount of data was collected and regarded sufficient to answer the research aim. This point was reached and jointly decided among authors. At this stage, eighteen nutrition leader participants had been recruited and most of them interviewed, with data analysis ongoing.

### The interviews

Semi-structured interviews were conducted individually face-to-face in a calm, mutually agreed space, primarily at the participant’s workplace. The interviews, guided by an interview guide specifically developed for this study, were audio recorded and conducted by the first author, a registered dietitian with foodservice and clinical experience, as part of her doctoral studies. The semi-structured interview guide questions aimed to capture the implementation process sequentially. Thus, the guide covered approaches, strategies, activities, challenges, and the different contexts involved, and was constructively guided by the i-PARIHS framework [48]. An English language version of the interview guide is displayed in Table 1. The interview guide was piloted with a manager from a similar context and experience, resulting in a modification of the sequence of questions (data from the pilot interview was not included in the final analysis). The guide allowed flexibility in the questions, including follow-ups, depending on the evolving content during the interview, which is in accordance with qualitative interview standards [54]. At the end of each interview, the interviewer reviewed and confirmed the main points discussed with the participant [55]. Information regarding the characteristics of the participants and the hospital settings was documented. Notes of interest, phrases, and concepts were recorded to enable analysis [56]. Interviews were automatically transcribed using NVivo 13 (2020 R1) [57] and its tool for transcription, and the first author reviewed and corrected the verbatim transcripts to ensure coherency with audio-recordings of interviews.

**Table 1:** Interview guide on hospital food and meal improvement initiatives: developed to capture the implementation process sequentially

**INTRODUCTORY QUESTION** I have asked you for this interview because of your ongoing… Would you like to tell us a little more about what you do? ***Examples of follow-up question for support:*** *Describe what is new or innovative about your work.* *Tell us more about the improvement work. What new initiatives have you taken or are currently doing?*
**MAIN QUESTIONS**
**1. WHAT MAKES THE CHANGE PROCESS TAKE PLACE?**
Purpose: Why is this happening right now? Why is the change needed? For what reason? ***Examples of follow-up questions for support: *** *Open follow-up question about the guidelines if it is not addressed of its own accord.* *How come you are doing this? What is the purpose? What is the reason for the improvement work? Why is it needed? Have the operations been affected by the National Food Agency's new national guidelines for meals in hospitals? If so, how has it affected them?*
**2. WHO CONTROLS THE WORK?**
Who has initiated the work, is responsible, decides, governance and management functions? ***Examples of follow-up questions for support:*** *Who is the initiator of the improvement work? Who has been involved in designing the improvement work? Who decides on the improvement work? *
**3. WHERE IS THE CHANGE IMPLEMENTED AND LIMITATIONS?**
Involved organisations/units/wards, partners, time frames and resources ***Examples of follow-up questions for support:*** *Which units within the hospital are involved or affected by the improvement work? Are there any other organisations or partners? How long has the improvement work been going on, and what does the time perspective look like in the future? Are there any time frames? What has the improvement work looked like in terms of resources? Has it been necessary to request money for this, new services, or similar? Has the improvement work been done within existing resources? Have you defined any limitations?*
**4. HOW DOES THE IMPLEMENTATION AND POSSIBLE IMPLEMENTATION STRATEGY TAKE PLACE?**
What approach, how, where, if a specific model or system was used ***Example of a follow-up question for support:*** *Has any specific model or system been used in the implementation?*
**5. ORGANISATIONAL CHALLENGES AND OPPURTUNITIES**
Lessons and experiences, what could have been done differently, what has worked well/less well? ***Example of a follow-up question for support:*** *Now that you have come a long way in this work, what could you have done differently?*
** 6. MONITORING AND EVALUATION**
The work ahead, what to do next, division of responsibilities, will the work be followed up and/or evaluated, and if so, what and how, if outcome measures or similar have been used? ***Example of a follow-up question for support:*** *Have outcome measures or similar been used to measure the results of your improvement work, and if so, which and how have these measures been followed and evaluated, and the results used?*
**CLOSING QUESTION** Is there anything in this work, in relation to what we have talked about, that you would like to add?
**SUMMARY**

### The analysis

The analysis followed Braun & Clarke’s six steps of thematic analysis [58], in which a flexible reflexive method is not tied to a specific theory or epistemology [59]. Firstly, the analysis consisted of repeated readings of transcripts by the first author to enable familiarisation with the content, with significant contributions from the last author. In the second step, using NVivo 13 (2020 R1) [57], the first author coded all the transcripts. The coding process employed an inductive approach, relying on the data and being responsive to the occurrences reported in the text. All authors were involved in checking transcripts and reviewing codes several times during the analysis process to ensure that the codes identified were comprehensive and reflected the data. The third step in the analysis involved mapping and identifying subthemes and themes, as well as pairing and grouping the most prevalent and pivotal codes, which included a set of themes suggested by the first author. The themes underwent refinement in the fourth step of thematic analyses through iterative author discussions. The i-PARIHS framework [48] was used as a lens in this step. The use of a theoretical framework when analysing and interpreting qualitative data within the field of multidisciplinary research in healthcare is suggested by, for example, Gale et al. [60]. Moreover, a theoretical framework has been claimed to be specifically useful in relation to implementation studies [61, 62]. Subsequently, employing a theoretical framework as a lens, the analytical process transitioned to a deductive approach beyond the initial data-driven and inductive stage, allowing the theory to guide the analysis. Considering this, extra attention was paid to key dimensions of i-PARIHS identified in the data set: facilitation activities, facilitators, and the specific context. The analysis process continued until the final themes were conclusively determined in the fifth step and then clearly defined in the sixth and final step when writing up the results. The results are presented thematically with examples of quotes translated from Swedish to English by a professional translator, considering any cultural gaps. The results are reported in accordance with the consolidated criteria for reporting qualitative research (COREQ) checklist [63].

### The researchers’ reflexivity

Researchers’ reflexivity and acknowledgement of potential biases were supported through multiple rounds of discussion among all authors when interpreting findings and providing feedback on analysis drafts. Integrating diverse perspectives minimised the risk of any single viewpoint dominating the results. Additionally, the researchers brought clinical and research expertise in nutritional care, dietetic practice, foodservice management, qualitative and implementation science research, patient-centred care, and public health nutrition. Such diverse backgrounds enabled a comprehensive and nuanced approach to data analysis and interpretation. As all authors (and participants) belong to the field of food, nutrition, and dietetics, their common understanding may result in some aspects being taken for granted. Furthermore, any previous professional relationships between participants and researchers were maintained professionally throughout the entire research process.

### The ethical considerations

The study was approved by the Swedish Ethical Review Authority (Dnr 2022-01110-01). Participants received information about the study before providing verbal and written consent. Personal data were omitted from the text to ensure secure data sharing, and storage occurred in a safe digital space authorised by Uppsala University. Quotes and characteristics are presented in a manner that ensures confidentiality. As this study involved human participants, the relevant ethical guidelines and regulations, such as the Declaration of Helsinki, were followed.

## Results

### The background characteristics

The study included eighteen participants, all of whom were strongly involved in improvement initiatives at different hospitals throughout Sweden. Individual interviews lasted 60–90 min, generating over 24 h of audio data. Their nutrition leader roles were defined either as foodservice managers, development managers for specific food and meal assignments, or as clinical dietitians– the latter two involving informal leadership. The majority of participants were female and had considerable work experience in the field. The improvement initiatives participants were involved in were either fairly recently introduced or ongoing for some time. All initiatives covered the same phenomenon: the provision of food and meals for hospital inpatients. Although they varied in scope, being planned either for a single hospital, a regional healthcare service, or at the national level, these efforts are all referred to as ‘initiatives’ in this paper.

Some participants were involved in multiple initiatives, while others took part in the same ones. In total, twelve Regions were included, representing forty-five hospitals, as some initiatives spanned several hospitals within a Region. More comprehensive background characteristics of participants and settings are shown in Table 2.

**Table 2:** Background and setting characteristics of the 18 participants

***Background characteristics***		*n*/yrs.
*Age*	Mean	47 yrs.
	Range	32–64 yrs.
*Gender*	Female	16
	Male	2
	Non-binary	0
*Experience in the field*	Median	10 yrs.
	Range	1–37 yrs.
*Experience in current position*	Median	3 yrs.
	Range	1–13 yrs.
*Position*	General manager	9
	Development manager	7
	Clinical dietitian	2
*Highest level of education*	Masters/PhD	6
	Bachelors	10
	Academic/other post-secondary	2
*Affiliation*	Service division	12
	Clinical division	6

***Setting characteristics***		***n***
*Hospital coverage*	Several	12
	Single	6
*Foodservice operator*	Public	10
	Private	7
	Public and private	1
*Foodservice staff in wards*	Yes	15
	No	3
*Lunch portions/day*	Median	600
	Range	150–11000
*Foodservice production*	Cook serve	3
	Cook chill	9
	Combination of several	6

To improve the provision of food and meals for hospital inpatients, the initiatives focused on multiple aspects, such as menu interventions and changes in production, distribution and meal ordering style. Some initiatives involved changes to the mealtime situation and the dining room environment, while most were focused on service performance, flexibility in meal ordering, and patient choices. However, this paper does not focus on the content of these initiatives and associated changes. Instead, it focuses on how to implement them and the facilitation activities for their realisation. Furthermore, the facilitation activities within these improvement initiatives, as reported by the participants’ experiences, are presented through categorisation into different themes.

### The themes

**Fig. 1:**
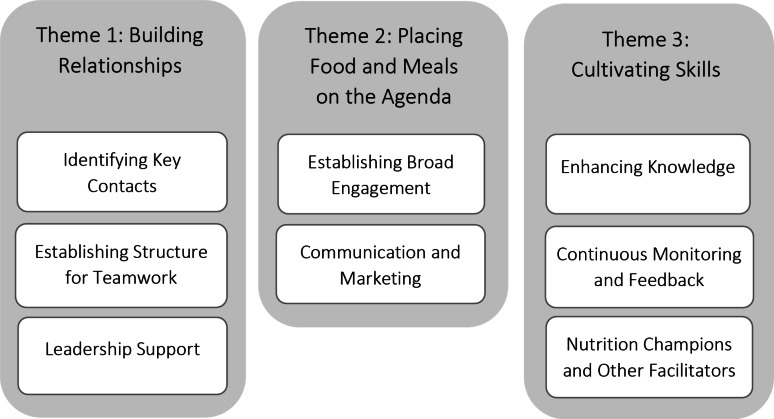
Overview of themes and subthemes

The analysis resulted in three themes: (1) ‘Building Relationships’, (2) ‘Placing Food and Meals on the Agenda’, and (3) ‘Cultivating Skills’. Barriers and several challenges emerged in implementing changes, necessitating ongoing and, in some cases, continuous efforts across various facilitation activities. ‘Building Relationships’ involves ‘Identifying Key Contacts’, ‘Establishing Structures for Teamwork’ and ‘Leadership Support’ amongst the many professionals and departments involved. ‘Placing Food and Meals on the Agenda’ focuses on gaining attention and recognition for improvement initiatives through ‘Establishing Broad Engagement’ and ‘Communication and Marketing’. ‘Cultivating Skills’ comprises facilitation activities for developing the competence needed and thereby include ‘Enhancing Knowledge’, ‘Continuous Monitoring and Feedback’, and ‘Nutrition Champions and Other Facilitators’. An overview of themes and subthemes is available in Fig. 1.

### Theme 1: Building relationships

All participants described the responsibility for food and meals for inpatients, and the performance of associated tasks, as being divided between the service division and the clinical division within the hospital. To implement change, it was necessary to build relationships through identifying key contacts, establishing structure for teamwork and leadership support.

#### Identifying key contacts

Building relationships necessitated identifying key contacts and crossing boundaries, to involve all relevant professional groups assigned food and meal tasks, representing both the service division (foodservice professionals) and the clinical division (clinical professionals). Further relationships were also required with several departments within each division and decision-makers at different hierarchical levels, including politicians at the regional level and managers high up in the hospital hierarchy. Participants particularly emphasised the importance of identifying key contacts within each division due to the involvement of several decision-makers with a mandate to implement changes, e.g., managers at different levels:

*Lobby in the right places; the Region is an organisational labyrinth. You have to find the right people and the right decision-makers. (Participant 15)*

The processes in hospitals for the provision of food and meals for inpatients were described as extensive, spanning from policy-making to meal-serving on the wards, making their structure difficult to comprehend. Having an extensive overview, including a broad outreach, was deemed crucial but perceived as challenging. This was eased by prior engagement, as well as personal connections and associates, enabling the creation of contact points for the participants and others to build relationships.

*I can change into hospital clothes and go to all the wards and I can make these contacts*,* which is really great… But the foodservice department has also been involved and used their contacts*,* so then you get a bit of an understanding of how it works at an operational level and you gain respect for that. (Participant 1)*

#### Establishing structure for teamwork

Establishing structures for teamwork was necessary because many participants explained that food and meal-related issues, including associated tasks that needed to be addressed, were often dealt with in silos rather than through communication and collaboration across organisational boundaries. One frequently mentioned challenge was the tendency to, sometimes unwittingly, frame potential collaborative partners in terms of ‘us versus them’, which served to maintain these silos:

*I think a lesson to learn is that you can never be too skilled in communication and organisation*,* and it easily becomes an ‘us and them’ situation. ‘Us’ who work in the kitchen*,* ‘them’ who work on the ward*,* or ‘us’ on the ward*,* ‘them’ in the kitchen. But it’s difficult*,* you know. We’re all working for the same cause. It’s obvious*,* of course*,* but in everyday life it’s not always obvious*,* or maybe I shouldn’t say ‘but’*,* it’s just that it feels like maybe we should have teamed up more*,* maybe we should have met up more. (Participant 12)*

In addition, participants expressed that different professionals and decision-makers had varying missions and held diverse perspectives. Regardless of the participants’ own background and current position, decision-makers high up in the hierarchy were often referred to as ‘them’ and described as being primarily financially focused. It was also common for clinical professionals to be referred to as ’them’, even by participants affiliated with the clinical division. According to the participants, clinical professionals, who typically see food and meal tasks as basic duties, often rushed through these tasks and were resistant to changes that might require additional work.

*It’s also the case that they (clinical professionals responsible for meal ordering) want to do this very quickly*,* take the orders. They write down on slips of paper all the dishes in the fridge*,* and then they say… what they often say is*,* ‘do you want fish*,* huh*,* do you want fish or meat or chicken?’ It’s not actually like that*,* there are fantastic names for all the dishes*,* really… such delicious restaurant names and all*,* but they don’t have time… (Participant 4)*.

Depending on their own division, the participants referred to foodservice professionals as either ’us’ or ’them’ and described them as service-driven. Additionally, most participants, even those with a clinical background, expected clinical dietitians to engage in patient food and meals, and not solely in aspects concerning medical nutritional therapy. However, it was commonly perceived that the level of engagement of clinical dietitians in how the patients’ food is prepared and served is too limited. This perception was expressed by several participants, including those who had experience working as clinical dietitians.

*Many clinical dietitians don’t believe that what ends up on the plate is their responsibility. (Participant 13)*

Overcoming these challenges and establishing better structures for increased and effective collaboration in teams was described by participants as requiring many facilitation activities, all of which involved building relationships. Changes were primarily implemented through forming different teams for structured multidisciplinary teamwork. The teams varied in size and configuration, depending on the type of assignment, and the importance of including various competencies was emphasised. This underscores the need to involve employees from both divisions, including those from several departments, for collaborations within and between the two divisions.

*So*,* collaboration and communication across boundaries*,* within our organisation and within our department (service division) - we have many different parts. We must work together better. Then*,* we must work together better with the other departments (within the clinical division). Well*,* I actually think there should be some kind of group related to food and nutrition*,* a group that meets*,* with representatives from everywhere. (Participant 14)*

#### Leadership support

Leadership support at various levels, in addition to the leaders of specific changes within the improvement initiatives, was crucial for the coordination of work and effective teamwork. For example, it helped to allocate sufficient time for facilitation activities within the overall schedule through the redistribution of certain tasks to others while implementing the changes. Participants appreciated this prioritisation, achieved through leadership support and active engagement. Demonstrating a genuine interest and respect for each other’s work was regarded as valuable, contributing to well-functioning relationships. One participant specifically stressed the importance of an inclusive leadership approach that embraces diverse opinions to further promote collaboration:

*Well*,* I believe that it’s mostly about a process of change*,* a significant part of working for change is having a different type of leadership where you invite people to be involved and share their opinions and thoughts*,* and that it doesn’t necessarily have to be our way that is applied. It’s about inviting people to join in… (Participant 16)*.

Connecting individuals at the same decision-making level, with leadership support, helped to mitigate power imbalances, enabling relationship-building and the implementation of changes. This was evident in meetings regarding the improvement initiatives and their associated changes. In addition, the facilitation activity of leadership support and coordination of work meetings helped to bridge the gap between the service and clinical divisions, both of which play significant roles in the provision of food and meals for inpatients. One participant, who coordinated meetings between professionals working at similar levels but representing different divisions, shared the following:

*And it’s very effective. Because when our employees (foodservice professionals) who are involved in food preparation meet those (clinical professionals) who serve the meals. Suddenly*,* you can make things happen in less than two days… It goes so smoothly*,* and it becomes so easy when you connect them*,* I mean people at the right level. (Participant 5)*

### Theme 2: Placing food and meals on the agenda

All participants stressed the importance of placing food and meals on the agenda to ensure support for changes to be implemented. In this context, ‘placing on the agenda’ means attracting attention and gaining recognition about an issue. It includes establishing broad engagement and communication and marketing.

#### Establishing broad engagement

Facilitation activities, all of which served in placing food and meals on the agenda, were directed towards the entire organisation by addressing several different groups: decision-makers, clinical professionals, foodservice professionals, participants’ own working groups within the improvement initiatives, and patients. All of these groups were regarded as important to reach for establishing a broad engagement, as food and meal issues impact and concern everyone.

Some activities aimed at placing food and meals on the agenda were undertaken early in the process of implementing changes, while others were performed later and recognised as ongoing work. However, attempts at establishing a broad engagement for placing food and meals on the agenda were often described as challenging. Participants noted that within the hospital setting, the food and meal service was regarded as a low priority:

*Yeah*,* but it is what it is*,* its low priority*,* a necessary evil in some way. I mean*,* then you just haven’t understood the importance of the basic thing with food*,* that it can make such a difference. It’s medications and pills*,* that’s what gets a lot of attention. (Participant 11)*

The view that food and meals are of low status emerged as a common and normalised perception in the interviews. Participants talked about how they constantly worked on establishing broad engagement in order to place food and meals on the agenda by drawing attention to the critical role of food in healthcare. The importance of food was emphasised by making comparisons to oral nutritional supplements:

*So*,* we work with it (the view of food and meals) everywhere*,* to the point where food is part of*,* I wouldn’t say the whole nutrition chain*,* but the whole thing with getting the patient to recover. It doesn’t start with nutritional products; we should start with the step before. The most important thing*,* or the best thing*,* is eating regular food before being given oral nutritional supplements*,* before getting on that ladder. But that’s it*,* we don’t have that at all*,* and food has been regarded as*,* you know*,* it’ll sort itself out*,* it’ll come. (Participant 17)*

#### Communication and marketing

Placing food and meals on the agenda involved general communication and marketing as facilitation activities, to address concerns highlighted during networking and interactions with diverse groups. The issues raised and the communication carried out were adapted to the specific group. For instance, when speaking with decision-makers, hospital food and meals were described as a patient safety issue:

*But it’s also a patient safety issue*,* and this concerns undernutrition and the risk of patients becoming undernourished and their recovery*,* and all of this*,* you know. If they don’t get food*,* there is an increased risk of readmission or longer hospital stays and all that. So*,* there are always many perspectives regarding meals*,* the issue of meals. (Participant 4)*

Some participants even described marketing efforts to reach out to hospital visitors and the general public to gain further recognition of food and meal issues. This involved connecting with various platforms and professional groups on social media and seeking publicity in the press.

### Theme 3: Cultivating skills

‘Cultivating skills’ represents facilitation activities focused on developing the competence necessary to achieve change by enhancing knowledge, continuous monitoring and feedback, as well as through nutrition champions and other facilitators.

#### Enhancing knowledge

Activities for enhancing knowledge, as experienced by several participants, were primarily educational in nature, following conventional methods and practices, and directed towards both clinical and foodservice professionals. They provided information and instructions about task performance and the necessary knowledge for implementing changes. They included conducting specific courses and workshops, using written documents, videos, and oral presentations. Participants also described activities that aimed to enhance familiarity with the food system they planned to implement. This was achieved by distributing free menu samples during meetings with different professionals, patients, and decision-makers from various departments, as well as showcasing meals.

‘Cultivating skills’ also involves facilitation activities to gain knowledge and an understanding of the implementation context. Participants described insights they collected from current practices through observations and from feedback given by those affected by the changes, including clinical and foodservice professionals, as well as patients. Sometimes, this included empathising with those concerned, both professionals and patients, through simulating a patient situation. This was perceived as useful, contributing to increased understanding and serving as teaching examples during the implementation work. Additionally, pilot projects served as ambassadors, setting examples to be learned from and used in the broader introduction of change and future implementations:

…*based on what you’ve learned*,* on the pilot wards*,* because that’s where you’ve learned it*,* and you’ve run out of steam a number of times trying to structure and build a model*,* how should we do this? Everything from changing working processes for the goods departments to how we should inform healthcare professionals? At what stage should it happen? How should we work with it to make it as smooth as possible for you? (Participant 17)*

#### Continuous monitoring and feedback

To ensure the suitability of improvement initiatives while cultivating skills, associated changes were consistently monitored, and feedback was regularly sought. Continuous monitoring and feedback helped to outline potential challenges that required attention. According to some participants, feedback played a crucial role in determining whether adjustments to changes were necessary or if further skills training was warranted. Consequently, feedback often led to modifying implemented changes and providing information and instructions on updates. In addition, continuously collected feedback assisted in tailoring changes, ensuring they were adapted to the specific context. Patient feedback, representing diverse care contexts in terms of diseases and symptoms, was regarded as valuable and obtained through patient questionnaires or input from professionals involved in serving meals. Clinical professionals acted as gatekeepers for obtaining patient feedback, especially from vulnerable and severely ill patients, a role that was particularly emphasised. Various activities, such as communicating with disease-specific patient associations and conducting interviews with individual patients, were used to gather this feedback. Furthermore, some participants mentioned the importance of reviewing what to ask patients and using broad open-ended questions to receive valuable feedback:

*What is important to them (the patients) and how can we get them to eat the food we serve? (Participant 13)*

These types of questions were regarded as having the potential to contribute to a deeper understanding of patient perceptions of food and their meal situations. All participants stressed the importance of patient involvement. For instance, obtaining valuable feedback from patients was seen as a way to identify the skills needed for improvement, and subsequently, to cultivate those skills among the staff.

#### Nutrition champions and other facilitators

During the process of implementing changes, facilitation activities focused on cultivating skills through different professionals acting as facilitators. These facilitators, nutrition champions and other facilitators, provided gentle reminders until new habits became routine. Functioning as clinical teachers, they contributed to implementing changes through their continuous efforts in everyday practice, creating learning opportunities that cultivated the necessary skills over time. According to participants, physicians, due to their high status, were particularly suitable as facilitators, and care managers and speech therapists were also mentioned as playing important roles. The primary facilitators were nutrition champions, i.e., clinical dietitians and foodservice professionals, who were regarded as experts in food and nutrition. Their ability to cultivate skills when engaging in ward meetings, stimulating learning dialogues, and encouraging the adoption of new practices was noted by several participants. The general perception was that such facilitators contributed to ongoing improvements, thereby enabling lasting change.

## Discussion

This paper explores nutrition leaders’ experiences of implementing changes, specifically the facilitation activities used to improve food and meal provision for hospital inpatients. These activities are categorised into ‘Building Relationships’, ‘Placing Food and Meals on the Agenda’, and ‘Cultivating Skills’, encompassing various approaches. These collaborative, communicative, educational and learning activities aimed to integrate changes in the provision of food and meals into the broader clinical framework. Barriers and resistance in clinical settings made implementing changes challenging, necessitating integrative facilitation activities. This involved working across organisational and relational boundaries, establishing multilevel collaborative structures, and adopting context-specific communication and educational support systems. Importantly, these facilitation activities also fostered an inclusive feedback culture.

The findings in this paper align with the i-PARIHS framework [49], which emphasises collaborative, communicative, and educational activities in facilitation, contributing to structural teamwork and knowledge. In this paper, we provide in-depth knowledge about the approaches and the actions of facilitators in the context of hospital food and meal provision for inpatients. Our findings imply that the framework is a useful guide for exploring the facilitation of implementing changes within our research context.

Furthermore, in line with i-PARIHS, facilitation activities enhancing knowledge and adopting various systems for continuous monitoring and feedback acknowledged the experiences and perspectives of different professionals and patients. However, such an approach, including the inner context and the recipients most affected by change, as described by i-PARIHS, are important aspects that appeared underutilised and overlooked among participants. This reveals gaps and opportunities for improvement, such as increased and actual professional and patient involvement in line with other co-design approaches. Additionally, there is an increasing body of literature on learning health systems including co-design approaches to support successful and sustainable change. Studies involve healthcare leaders [64] and initiatives for vulnerable populations [65], which is similar to our research. Moreover, adequate organisational prerequisites, such as sufficient time, are described as crucial for the successful implementation of improvement initiatives within nutrition and mealtime care [66–69] and healthcare overall [70, 71]. Our findings support the significance of having these organisational prerequisites in place, as they address the challenges described by participants, such as gaining attention and recognition for improvement initiatives within a busy clinic and divided work environment. This further highlights gaps and opportunities for improvement.

Changing food and meal practices at ward level appeared to be challenging, as evidenced by the practice of rushing through these tasks, which was previously attributed to time constraints [69]. However, according to the participants’ descriptions, it was the seemingly normalised view that food and meals were a low priority, rather than time constraints, that led the staff to rush through these tasks. The low priority appeared to be related to knowledge gaps, as seen in other studies [19, 72]. Additionally, there was a lack of awareness of each other’s disciplines, stemming from working in silos, and ‘us’ versus ‘them’ communication, which marginalised food and meals as issues outside the clinical agenda. Nevertheless, participants described a significant shift in culture when forming teams and building relationships across divisional boundaries. This underscores the importance of identifying barriers prior to implementing changes in improvement initiatives, emphasising feedback as a continuous facilitation activity. Such contextual awareness further aligns with implementation and improvement initiatives emphasised by others [27, 28, 73, 74].

Moving away from silos involved engaging in multidisciplinary teamwork, which presupposes active participation and information sharing. Multidisciplinary teamwork, highly valued by participants and essential within food and meal provision [16, 75, 76], provides relevant expertise through combined competencies, pushing work forward and contributing to consensus. Our findings also highlight the role of nutrition champions [28, 74, 77] as facilitators offering regular support in everyday practice.

Continuous peer support by facilitators indicates the enhancement of leadership that adopts an inclusive approach towards those involved, incorporating an empathetic element. Shared decision-making, a feedback culture, and pilot tests for context-tailoring further reinforce this approach. The shared points in these leadership approaches include consideration of the voices of those involved, both employees and patients, and nurturing social awareness in the workplace. Such leadership styles, which include empathy, are widely acknowledged in recent leadership literature [78]. They are associated with relationship-oriented leadership theories: relational [79], mindful [80], and transformational [81]. Change is inevitably accompanied by tensions, making it crucial to embrace diverse voices initiating and implementing changes for improvements [82]. Such an approach is particularly important in the provision of food and meals due to the differing viewpoints among professionals, which in this study ranged from service-driven to financially focused. Consequently, differing priorities and unrecognised work can create tensions that pose challenges in improvement initiatives and related activities involving various tasks.

In healthcare overall, promoting an organisational culture of psychological safety where everyone feels that they are heard and that their opinions count, functions as a driver for change [83, 84] and effective team performance [85, 86]. Similar findings were shown in the participants’ accounts of building relationships, where setting up a culture that includes a power balance between the professionals involved was considered more fruitful for collaboration. Within a hierarchical organisation, such as a hospital, it is crucial to achieve a culture of safety where everyone can express their opinions equally [84]. A psychological safety culture further enables change across different organisational cultures [87]. This is particularly relevant to hospital food and meal provision, which combines both service and clinical cultures.

This study has strengths and limitations. One limitation pertains to the participants’ backgrounds, as few clinical professionals were interviewed. In addition, the focus on nutrition leaders meant that practical user experiences related to food and meal tasks and the impact of implementing changes were not captured. Future research within this field could benefit from interviewing care managers and professionals with direct user experience in implementing change, as well as patients. Interviewing patients with user-experience in future research would capture their insights on implemented changes and provide valuable in-depth feedback on implementation success for continues improvement. User experience refers to individuals who typically implement changes and represent the end-stage in the process, the inner-context in the organisation according to i-PARIHS, which contrasts with the perspectives of those leading change. Nevertheless, the strengths of the study lie in its in-depth insights into leadership approaches within the complex, multilevel context of food and meal provision for hospital inpatients. Furthermore, it highlights various facilitation activities in implementing changes across multiple improvement initiatives. Another strength is the participation of several individuals involved in similar, and sometimes the same, food and meal improvement initiatives [88]. Interviewing multiple participants about the same phenomenon enhances the study by incorporating diverse perspectives [89]. Including participants from a broad geographical range, combined with various facilitation activities, adds depth and differentiation to the findings. This enhances the transferability of the study findings, particularly to developed countries with healthcare systems similar to Sweden’s.

### Implications for policy and future work

Based on the study findings, several implications for policy and future work are provided. Implementing changes to improve food and meal provision for hospital inpatients requires crossing organisational and relational boundaries, considering the roles of respective professionals. Facilitation activities through active leadership, including ongoing support, monitoring, and feedback, are crucial for implementing change. Utilising nutrition champions as facilitators, who guide change and provide gentle reminders until new routines become everyday practice, is recommended. A list of recommended facilitation activities for leaders to support implementation success is provided in Table 3.

**Table 3:** Recommended facilitation activities to leaders to support implementation success

**Engage in Teamwork**
Leaders are recommended to value and engage in inter- and multidisciplinary teamwork, considering the roles of respective professionals.
**Establish Effective Feedback Systems**
Leaders are recommended to establish feedback systems for appropriate supervision of work.
**Appoint facilitators**
Leaders are recommended to act as ambassadors for change by appointing individuals or groups to facilitator roles.

## Conclusions

‘Building Relationships’, ‘Placing Food and Meals on the Agenda’, and ‘Cultivating Skills’ are key to improving food and meal provision for hospital inpatients. Based on the experiences of nutrition leader participants and our analysis, these keys comprise multiple facilitation activities. Collaboration between foodservice and clinical professionals, along with the dissemination of knowledge, appears to be important for implementing changes. Active leadership supports successful implementations by providing structured approaches, including feedback systems, and by contributing to the recognition of improvement initiatives, according to experiences shared during interviews. These findings may guide policy and future work, including improvement initiatives within hospitals, specifically regarding food and meals.

## Data Availability

The raw data supporting the conclusions in this article are not readily available because no permission was given by the participants for anyone to have the raw data except the principal investigators. Requests to access the datasets should be directed to emma.wilandh@ikv.uu.se.

## References

[1] Downer S, Berkowitz SA, Harlan TS, Olstad DL, Mozaffarian D. Food is medicine: actions to integrate food and nutrition into healthcare. BMJ. 2020;369:m2482. 10.1136/bmj.m2482. 32601089 10.1136/bmj.m2482 PMC7322667

[2] Zhao Y, Zhang Y, Hao Q, Ge M, Dong B. Sarcopenia and hospital-related outcomes in the old people: a systematic review and meta-analysis. Aging Clin Exp Res. 2019;31(1):5–14. 10.1007/s40520-018-0931-z. 29549649 10.1007/s40520-018-0931-z

[3] Wells JC, Sawaya AL, Wibaek R, Mwangome M, Poullas MS, Yajnik CS, Demaio A. The double burden of malnutrition: aetiological pathways and consequences for health. Lancet. 2020;395(10217):75–88. 10.1016/s0140-6736(19)32472-9. 31852605 10.1016/S0140-6736(19)32472-9 PMC7613491

[4] Tarantino S, Hiesmayr M, Sulz I, nDay working group. nutritionDay Worldwide Annual Report 2019. Clin Nutr ESPEN. 2022;49:560–667. 10.1016/j.clnesp.2022.01.001. 35623868 10.1016/j.clnesp.2022.01.001

[5] Kruizenga H, van Keeken S, Weijs P, Bastiaanse L, Beijer S, Huisman-de Waal G, Jager-Wittenaar H, Jonkers-Schuitema C, Klos M, Remijnse-Meester W, et al. Undernutrition screening survey in 564,063 patients: patients with a positive undernutrition screening score stay in hospital 1.4 d longer. Am J Clin Nutr. 2016;103(4):1026–32. 10.3945/ajcn.115.126615. 26961930 10.3945/ajcn.115.126615

[6] Tappenden KA, Quatrara B, Parkhurst ML, Malone AM, Fanjiang G, Ziegler TR. Critical role of nutrition in improving quality of care: an interdisciplinary call to action to address adult hospital malnutrition. J Acad Nutr Diet. 2013;113(9):1219–37. 10.1016/j.jand.2013.05.015. 23871528 10.1016/j.jand.2013.05.015

[7] Osman NS, Md Nor N, Md Sharif MS, Hamid SBA, Rahamat S. Hospital food service strategies to improve food intakes among inpatients: A systematic review. Nutrients. 2021;13(10). 10.3390/nu13103649. 10.3390/nu13103649 PMC8537902 34684649

[8] Cheung G, Pizzola L, Keller H. Dietary, food service, and mealtime interventions to promote food intake in acute care adult patients. J Nutr Gerontol Geriatr. 2013;32(3):175–212. 10.1080/21551197.2013.809673. 23924254 10.1080/21551197.2013.809673

[9] Munk T, Bruun N, Nielsen MA, Thomsen T. From evidence to clinical practice: positive effect of implementing a Protein-Enriched hospital menu in conjunction with individualized dietary counseling. Nutr Clin Pract. 2017;32(3):420–6. 10.1177/0884533616688432. 28145792 10.1177/0884533616688432

[10] Aggarwal M, Grady A, Desai D, Hartog K, Correa L, Ostfeld RJ, Freeman AM, McMacken M, Gianos E, Reddy K, et al. Successful implementation of healthful nutrition initiatives into hospitals. Am J Med. 2020;133(1):19–25. 10.1016/j.amjmed.2019.08.019. 31494109 10.1016/j.amjmed.2019.08.019

[11] Neaves B, Bell JJ, McCray S. Impact of room service on nutritional intake, plate and production waste, meal quality and patient satisfaction and meal costs: A single site pre-post evaluation. Nutr Diet. 2022;79(2):187–96. 10.1111/1747-0080.12705. 34609060 10.1111/1747-0080.12705

[12] Doorduijn AS, van Gameren Y, Vasse E, de Roos NM. At your Request((R)) room service dining improves patient satisfaction, maintains nutritional status, and offers opportunities to improve intake. Clin Nutr. 2016;35(5):1174–80. 10.1016/j.clnu.2015.10.009. 26608525 10.1016/j.clnu.2015.10.009

[13] Dijxhoorn DN, VE IJ-H, Wanten GJA, van den Berg MGA. Strategies to increase protein intake at mealtimes through a novel high-frequency food service in hospitalized patients. Eur J Clin Nutr. 2019;73(6):910–6. 10.1038/s41430-018-0288-6. 30135550 10.1038/s41430-018-0288-6

[14] Mortensen MN, Larsen AK, Skadhauge LB, Høgsted RH, Beermann T, Cook ME, Rasmussen HH, Mikkelsen BE, Holst M. Protein and energy intake improved by in-between meals: an intervention study in hospitalized patients. Clin Nutr ESPEN. 2019;30:113–8. 10.1016/j.clnesp.2019.01.007. 30904210 10.1016/j.clnesp.2019.01.007

[15] Munk T. Strategies to optimise nutritional practice for patients at nutritional risk– With special emphasis on hospital food and individual dietary counselling. Aalborg: Aalborg Universitetsforlag; 2015. 10.5278/vbn.phd.med.00004.

[16] Ross LJ, Mudge AM, Young AM, Banks M. Everyone’s problem but Nobody’s job: staff perceptions and explanations for poor nutritional intake in older medical patients. Nutr Diet. 2011;68(1):41–6. 10.1111/j.1747-0080.2010.01495.x.

[17] Committee of Ministers. Resolution ResAP(2003)3 on food and nutritional care in hospitals. Strasbourg: Council of Europe; 2003. Available from: https://rm.coe.int/09000016805de855. Accessed 3 Mar 2025.

[18] Trang S, Fraser J, Wilkinson L, Steckham K, Oliphant H, Fletcher H, Tzianetas R, Arcand J. A Multi-Center assessment of nutrient levels and foods provided by hospital patient menus. Nutr. 2015;7(11):9256–64. 10.3390/nu7115466. 10.3390/nu7115466 PMC4663594 26569294

[19] Laur C, Marcus H, Ray S, Keller H. Quality nutrition care: measuring hospital staff’s knowledge, attitudes, and practices. Healthc (Basel). 2016;4(4):79. 10.3390/healthcare4040079. 10.3390/healthcare4040079 PMC5198121 27775604

[20] Braithwaite J, Churruca K, Long JC, Ellis LA, Herkes J. When complexity science Meets implementation science: a theoretical and empirical analysis of systems change. BMC Med. 2018;16(1):63. 10.1186/s12916-018-1057-z. 29706132 10.1186/s12916-018-1057-z PMC5925847

[21] Sarkies M, Robinson S, Ludwick T, Braithwaite J, Nilsen P, Aarons G, Weiner BJ, Moullin J. Understanding implementation science from the standpoint of health organisation and management: an interdisciplinary exploration of selected theories, models and frameworks. J Health Organ Manag. 2021;35(7):782–801. 10.1108/jhom-02-2021-0056.

[22] Melder A, Robinson T, McLoughlin I, Iedema R, Teede H. An overview of healthcare improvement: unpacking the complexity for clinicians and managers in a learning health system. Intern Med J. 2020;50(10):1174–84. 10.1111/imj.14876. 32357287 10.1111/imj.14876

[23] Olufson HT, Young AM, Green TL. The delivery of patient centred dietetic care in subacute rehabilitation units: A scoping review. J Hum Nutr Diet. 2022;35(1):134–44. 10.1111/jhn.12940. 34370342 10.1111/jhn.12940

[24] Bell JJ, Young AM, Hill JM, Banks MD, Comans TA, Barnes R, Keller HH. Systematised, interdisciplinary malnutrition program for implementation and evaluation delivers improved hospital nutrition care processes and patient reported experiences - An implementation study. Nutr Diet. 2021;78(5):466–75. 10.1111/1747-0080.12663. 33817934 10.1111/1747-0080.12663

[25] Eliot KA, L’Horset AM, Gibson K, Petrosky S. Interprofessional education and collaborative practice in nutrition and dietetics 2020: an update. J Acad Nutr Diet. 2021;121(4):637–46. 10.1016/j.jand.2020.08.010. 33008786 10.1016/j.jand.2020.08.010

[26] Olufson H, Ottrey E, Young A, Green T. Opportunity, hierarchy, and awareness: an ethnographic exploration across rehabilitation units of interprofessional practice in nutrition and mealtime care. J Interprof Care 2023:1–10. 10.1080/13561820.2023.2243287 10.1080/13561820.2023.2243287 37587555

[27] Gerrish K, Laker S, Taylor C, Kennedy F, McDonnell A. Enhancing the quality of oral nutrition support for hospitalized patients: a mixed methods knowledge translation study (The EQONS study). J Adv Nurs. 2016;72(12):3182–94. 10.1111/jan.13085. 27485574 10.1111/jan.13085

[28] Cave D, Abbey K, Capra S. Food and nutrition champions in residential aged care homes are key for sustainable systems change within foodservices; results from a qualitative study of stakeholders. Nutr. 2021;13(10):3566. 10.3390/nu13103566. 10.3390/nu13103566 PMC8541169 34684566

[29] Holst M, Beermann T, Mortensen MN, Skadhauge LB, Lindorff-Larsen K, Rasmussen HH. Multi-modal intervention improved oral intake in hospitalized patients. A one year follow-up study. Clin Nutr. 2015;34(2):315–22. 10.1016/j.clnu.2014.05.001. 24874177 10.1016/j.clnu.2014.05.001

[30] Conchin S, Carey S. The expert’s guide to mealtime interventions - A Delphi method survey. Clin Nutr. 2018;37(6 Pt):1992–2000. 10.1016/j.clnu.2017.09.005. 29029892 10.1016/j.clnu.2017.09.005

[31] Calleja-Fernández A, Velasco-Gimeno C, Vidal-Casariego A, Pintor-de-la-Mazaa B, Frías-Sorianoc L, Villar-Taiboa R, García-Perisc P, Cano-Rodrígueza I, García-Fernándezb C, Ballesteros-Pomara MD. Impact of kitchen organization on oral intake of malnourished inpatients: A two-center study. Endocrinol Diabetes Nutr (Engl Ed). 2017;64(8):409–16. 10.1016/j.endien.2017.10.003. 10.1016/j.endinu.2017.05.003 28895536

[32] Kozica-Olenski S, Treleaven E, Hewitt M, McRae P, Young A, Walsh Z, Mudge A. Patient-reported experiences of mealtime care and food access in acute and rehabilitation hospital settings: a cross-sectional survey. J Hum Nutr Diet. 2021;34(4):687–94. 10.1111/jhn.12854. 33491875 10.1111/jhn.12854

[33] McCray S, Maunder K, Norris R, Moir J, MacKenzie-Shalders K. Bedside menu ordering system increases energy and protein intake while decreasing plate waste and food costs in hospital patients. Clin Nutr ESPEN. 2018;26:66–71. 10.1016/j.clnesp.2018.04.012. 29908685 10.1016/j.clnesp.2018.04.012

[34] Díez-Garicía RW, Zangiacomi E, Rodrigues de Oliveira Penaforte F, Cremonezi Japur C. Hospital nutritional care: propositions endorsed by the scientific community. Nutr Hosp. 2015;32(3):1353–61. 26319860 10.3305/nh.2015.32.3.9307

[35] Ranke TD, Mitchell CL, St George DM, D’Adamo CR. Evaluation of the balanced menus challenge: a healthy food and sustainability programme in hospitals in Maryland. Public Health Nutr. 2015;18(13):2341–9. 10.1017/S1368980014002936. 25543666 10.1017/S1368980014002936 PMC10335773

[36] Pullen K, Collins R, Stone T, Carter H, Sadler H, Collinson A. Are energy and protein requirements Met in hospital? J Hum Nutr Diet. 2018;31(2):178–87. 10.1111/jhn.12485. 28586107 10.1111/jhn.12485

[37] Laur C, Valaitis R, Bell J, Keller H. Changing nutrition care practices in hospital: a thematic analysis of hospital staff perspectives. BMC Health Serv Res. 2017;17(1). 10.1186/s12913-017-2409-7. 10.1186/s12913-017-2409-7 PMC5518103 28724373

[38] Ottrey E, Palermo C, Huggins CE, Porter J. A longitudinal ethnographic study of hospital staff attitudes and experiences of change in nutrition care. J Hum Nutr Diet. 2020;33(4):574–83. 10.1111/jhn.12734. 31989752 10.1111/jhn.12734

[39] Sorensen J, Fletcher H, Macdonald B, Whittington-Carter L, Nasser R, Gramlich L. Canadian hospital food service practices to prevent malnutrition. Can J Diet Pract Res. 2021;82(4):167–75. 10.3148/cjdpr-2021-013. 34286621 10.3148/cjdpr-2021-013

[40] Clinical Excellence Queensland. Foodservice best practice. Brisbane: Queensland health guideline; 2023. p. 1–13.

[41] The Swedish Food Agency. National guidelines for meals in hospitals. In: Nationella Riktlinjer för måltider På Sjukhus. Uppsala: Livsmedelsverket (SE); 2020.

[42] Thibault R, Abbasoglu O, Ioannou E, Meija L, Ottens-Oussoren K, Pichard C, Rothenberg E, Rubin D, Siljamäki-Ojansuu U, Vaillant MF, et al. ESPEN guideline on hospital nutrition. Clin Nutr. 2021;40(12):5684–709. 10.1016/j.clnu.2021.09.039. 34742138 10.1016/j.clnu.2021.09.039

[43] Skånberg E, Eriksson E. Mapping of meal activities in hospitals 2022 - Facts about public meals. *L 2022 Nr. 18: Kartläggning av måltidsverksamheten på sjukhus 2022 - Fakta om offentliga måltider.* In: *The Swedish Food Agency´s report series.* Edited by Agency TSF. Uppsala: Livsmedelsverket (SE); 2022:52.

[44] Wilandh E, Skinnars Josefsson M, Persson Osowski C, Mattsson Sydner Y. Better hospital foodservice– aspects highlighted in research published 2000–2023: A scoping review. Clin Nutr Open Sci. 2024;54:1–40. 10.1016/j.nutos.2024.01.001.

[45] Harvey G, Loftus-Hills A, Rycroft-Malone J, Titchen A, Kitson A, McCormack B, Seers K. Getting evidence into practice: the role and function of facilitation. J Adv Nurs. 2002;37(6):577–88. 10.1046/j.1365-2648.2002.02126.x. 11879422 10.1046/j.1365-2648.2002.02126.x

[46] Weinberg D. The philosophical foundations of constructionist research. In: Holstein JA, Gubrium JF, editors. *Handbook of constructionist research*. 1st ed. New York: Guildford Press; 2008. p. 13–39.

[47] Patton MQ. Qualitative research & evaluation methods: integrating theory and practice. 4th ed. Thousand Oaks: SAGE Publications, Inc; 2015.

[48] Harvey G, Kitson A. PARIHS revisited: from heuristic to integrated framework for the successful implementation of knowledge into practice. Implement Sci. 2016;11:33. 10.1186/s13012-016-0398-2. 27013464 10.1186/s13012-016-0398-2 PMC4807546

[49] Harvey G, Kitson A. Implementing Evidence-Based practice in healthcare, a facilitation guide. London & New York: Routledge Taylor & Francis Group; 2015.

[50] Williams C, van der Meij BS, Nisbet J, McGill J, Wilkinson SA. Nutrition process improvements for adult inpatients with inborn errors of metabolism using the i-PARIHS framework. Nutr Diet. 2019;76(2):141–9. 10.1111/1747-0080.12517. 30848058 10.1111/1747-0080.12517

[51] Mudge AM, McRae P, Young A, Blackberry I, Lee-Steere K, Barrimore S, Quirke T, Harvey G. Implementing a ward-based programme to improve care for older inpatients: process evaluation of the cluster randomised CHERISH trial. BMC Health Serv Res. 2023;23(1):668. 10.1186/s12913-023-09659-2. 37344776 10.1186/s12913-023-09659-2 PMC10283300

[52] Kvale S. Interviews. Thousand Oaks: Sage; 1996.

[53] Varpio L, Ajjawi R, Monrouxe LV, O’Brien BC, Rees CE. Shedding the Cobra effect: problematising thematic emergence, triangulation, saturation and member checking. Med Educ. 2017;51(1):40–50. 10.1111/medu.13124. 27981658 10.1111/medu.13124

[54] Kvale S, Brinkmann S. Interviews: learning the craft of qualitative research interviewing. 2nd ed. Los Angeles: SAGE; 2009.

[55] Gill P, Stewart K, Treasure E, Chadwick B. Methods of data collection in qualitative research: interviews and focus groups. Br Dent J. 2008;204(6):291–5. 10.1038/bdj.2008.192. 18356873 10.1038/bdj.2008.192

[56] Pontin D. Interviews. In: *The research process in nursing.* 4th edn. Cormack D. Eds. Oxford: Blackwell Science; 2000: 289–298.

[57] Lumivero. NVivo 13 (2020, R1). https://www.lumivero.com. 2022.

[58] Braun V, Clarke V. Using thematic analysis in psychology. Qual Res Psychol. 2006;3(2):77–101. 10.1191/1478088706qp063oa.

[59] Braun V, Clarke V. Thematic analysis: A practical guide. London: SAGE; 2022.

[60] Gale NK, Heath G, Cameron E, Rashid S, Redwood S. Using the framework method for the analysis of qualitative data in multi-disciplinary health research. BMC Med Res Methodol. 2013;13(117):1–8. 10.1186/1471-2288-13-117. 24047204 10.1186/1471-2288-13-117 PMC3848812

[61] Bamford C, Heaven B, May C, Moynihan P. Implementing nutrition guidelines for older people in residental care homes: a qualitative study using normalization process theory. Implement Sci. 2012;106(7). 10.1186/1748-5908-7-106. 10.1186/1748-5908-7-106 PMC3514214 23110857

[62] Laur C, Keller HH. Implementing best practice in hospital multidisciplinary nutritional care: an example of using the knowledge-to-action process for a research program. J Multidiscip Healthc. 2015;8:463–72. 10.2147/JMDH.S93103. 26491344 10.2147/JMDH.S93103 PMC4599637

[63] Tong A, Sainsbury P, Craig J. Consolidated criteria for reporting qualitative research (COREQ): a 32-item checklist for interviews and focus groups. Int J Qual Health Care. 2007;19(6):349–57. 10.1093/intqhc/mzm042. 17872937 10.1093/intqhc/mzm042

[64] Enticott J, Braaf S, Johnson A, Jones A, Teede HJ. Leaders’ perspectives on learning health systems: a qualitative study. BMC Health Serv Res. 2020;20(1):1087. 10.1186/s12913-020-05924-w. 33243214 10.1186/s12913-020-05924-w PMC7689994

[65] Siou K, Charles J, Convery C, Henderson T. Identifying older adults with changing care needs: a co-designed community-based screening tool… First North American Conference on Integrated Care, October 4–7, 2021, Toronto, Ontario. Int J Integr Care 2022;22:1–2. 10.5334/ijic.ICIC21321.

[66] Young A, Kozica-Olenski S, Mallan K, McRae P, Treleaven E, Walsh Z, Mudge A. Developing and validating a novel staff questionnaire to identify barriers and enablers to nutrition and mealtime care on hospital wards. Nutr Diet. 2023;80(4):389–98. 10.1111/1747-0080.12815. 37169361 10.1111/1747-0080.12815

[67] Nielsen LP, Thomsen KH, Alleslev C, Mikkelsen S, Holst M. Implementation of nutritional care in hospitals: A qualitative study of barriers and facilitators using implementation theory. Scand J Caring Sci. 2024;38(3):657–68. 10.1111/scs.13255. 38520146 10.1111/scs.13255

[68] Young AM, Byrnes A, Mahoney D, Power G, Cahill M, Heaton S, McRae P, Mudge A, Miller E. Exploring hospital mealtime experiences of older inpatients, caregivers and staff using photovoice methods. J Clin Nurs. 2024;33(5):1906–20. 10.1111/jocn.17009. 38284486 10.1111/jocn.17009

[69] Trinca V, Duizer L, Paré S, Keller H. Investigating the patient food experience: Understanding hospital staffs’ perspectives on what leads to quality food provision in Ontario hospitals. J Hum Nutr Diet. 2022;35(5):980–94. 10.1111/jhn.12964. 34786772 10.1111/jhn.12964

[70] Fulop NJ, Ramsay A. How organisations contribute to improving the quality of healthcare. BMJ. 2019;365:l1773. 10.1136/bmj.l1773. 31048322 10.1136/bmj.l1773 PMC6495298

[71] Batalden P. Getting more health from healthcare: quality improvement must acknowledge patient coproduction—an essay by Paul batalden. BMJ. 2018;362:k3617. 10.1136/bmj.k3617.

[72] Wong S, Derry F, Graham A, Grimble G, Forbes A. An audit to assess awareness and knowledge of nutrition in a UK spinal cord injuries centre. Spinal Cord. 2012;50(6):446–51. 10.1038/sc.2011.180. 22249328 10.1038/sc.2011.180

[73] Bergman C, Löve J, Hultberg A, Skagert K. Employees’ conceptions of coworkership in a Swedish health care organization. Nord J Work Life Stud. 2017;7(4):91–107. 10.18291/njwls.v7i4.102359.

[74] Yona O, Goldsmith R, Endevelt R. Improved meals service and reduced food waste and costs in medical institutions resulting from employment of a food service dietitian -a case study. Isr J Health Policy Res. 2020;9(1):5. 10.1186/s13584-020-0362-0. 32014056 10.1186/s13584-020-0362-0 PMC6998356

[75] Collins J, Huggins CE, Porter J, Palermo C. Factors influencing hospital foodservice staff’s capacity to deliver a nutrition intervention. Nutr Diet. 2017;74(2):129–37. 10.1111/1747-0080.12344. 28731638 10.1111/1747-0080.12344

[76] Ottrey E, Porter J, Huggins CE, Palermo C. Ward culture and staff relationships at hospital mealtimes in Australia: an ethnographic account. Nurs Health Sci. 2019;21(1):78–84. 10.1111/nhs.12559. 30105899 10.1111/nhs.12559

[77] Laur C, Bell J, Valaitis R, Ray S, Keller H. The sustain and spread framework: strategies for sustaining and spreading nutrition care improvements in acute care based on thematic analysis from the More-2-Eat study. BMC Health Serv Res. 2018;18(1):930. 10.1186/s12913-018-3748-8. 30509262 10.1186/s12913-018-3748-8 PMC6278089

[78] Tzouramani E. Leadership and empathy. In: Marques J, Dhiman S, editors. Leadership today: practices for personal and professional performance*.* Cham: Springer International Publishing; 2017. p. 197–216. 10.1007/978-3-319-31036-7.

[79] Jian G. From empathic leader to empathic leadership practice: an extension to relational leadership theory. Hum Relat. 2021;75(5):931–55. 10.1177/0018726721998450.

[80] Wibowo A, Paramita W. Resilience and turnover intention: the role of mindful leadership, empathetic leadership, and Self-Regulation. J Leadersh Organ Stud. 2021;29(3):325–41. 10.1177/15480518211068735.

[81] Rahman W. Transformational leadership and empathy: the impact of quality in the health care services in Kelantan, Malaysia. Int J Econom Bus Manag Stud. 2017;4(1):50–6. 10.20448/802.41.50.56.

[82] Mannion R, Davies HT. Cultures of silence and cultures of voice: the role of whistleblowing in healthcare organisations. Int J Health Policy Manag. 2015;4(8):503–5. 10.15171/ijhpm.2015.120. 26340388 10.15171/ijhpm.2015.120 PMC4529038

[83] Jamal N, Young VN, Shapiro J, Brenner MJ, Schmalbach CE. Patient safety/quality improvement primer, part IV: psychological Safety-Drivers to outcomes and Well-being. Otolaryngol Head Neck Surg. 2023;168(4):881–8. 10.1177/01945998221126966. 36166311 10.1177/01945998221126966

[84] Falcone M, Tokac U, Fish AF, Van Stee SK, Werner KB. Factor structure and construct validity of a hospital survey on patient safety culture using exploratory factor analysis (1). J Patient Saf. 2023;19(5):323–30. 10.1097/PTS.0000000000001126. 37144884 10.1097/PTS.0000000000001126

[85] Khan MF, Sewell MD, Alrawi A, Taif S, Divani K. Can a culture of team psychological safety and MDT proforma improve team performance and patient outcomes in spinal MDTs? Br J Neurosurg. 2022;1–5. 10.1080/02688697.2021.1967288. 10.1080/02688697.2021.1967288 35135402

[86] Rosenbaum L. Cursed by Knowledge — Building a culture of psychological safety. N Engl J Med. 2019;380(8):786–90. 10.1056/NEJMms1813429. 30786195 10.1056/NEJMms1813429

[87] Thorgren S, Caiman E. The role of psychological safety in implementing agile methods across cultures. Res Technol Manag. 2019;62(2):31–9. 10.1080/08956308.2019.1563436.

[88] Alvesson M, Torhell S-E. Interviews: conduct, interpretation, and reflexivity. In: Intervjuer: Genomförande, Tolkning och reflexivitet. Malmö: Liber; 2011.

[89] Brinkmann S, Kvale S. Doing interviews. 2nd ed. Los Angeles: SAGE; 2018.

